# Development of a Smoking Abstinence Self-efficacy Questionnaire

**DOI:** 10.1007/s12529-012-9229-2

**Published:** 2012-02-20

**Authors:** Viola Spek, Fieke Lemmens, Marlène Chatrou, Suzanne van Kempen, François Pouwer, Victor Pop

**Affiliations:** 1 Centre of Research on Psychology in Somatic diseases (CoRPS), Tilburg University, Tilburg, the Netherlands; 2 Department of Medical Psychology, Máxima Medical Centre Veldhoven, Veldhoven, the Netherlands; 3 Department of Medical Psychology, CoRPS - Centre for Research on Psychology in Somatic diseases, Tilburg University, PO Box 90153, 5000 Tilburg, the Netherlands

**Keywords:** Self-efficacy, Smoking, Abstinence, Psychometric Properties

## Abstract

**Background:**

**S**elf-efficacy beliefs are an important determinant of (changes in) health behaviors. In the area of smoking cessation, there is a need for a short, feasible, and validated questionnaire measuring self-efficacy beliefs regarding smoking cessation.

**Purpose:**

The purpose of this study is to investigate the psychometric properties of a six-item questionnaire to assess smoking cessation self-efficacy.

**Methods:**

We used longitudinal data from a smoking cessation study. A total of 513 smokers completed the Smoking Abstinence Self-efficacy Questionnaire (SASEQ) and questionnaires assessing depressive symptoms and motivation to quit smoking. After that, they set a quit date and attempted to stop smoking. One year after the quit date, smoking status of participants was assessed by self report. The psychometric properties of the SASEQ were studied and we investigated whether SASEQ scores predicted successful smoking cessation.

**Results:**

Factor analysis yielded one factor, with an Eigenvalue of 3.83, explaining 64% of variance. All factor loadings were ≥0.73. We found a Cronbach’s alpha of 0.89 for the SASEQ, low correlations for the SASEQ with depressive symptoms, and motivation to quit, indicating that self-efficacy is measured independently of these concepts. Furthermore, high baseline SASEQ scores significantly predicted smoking abstinence at 52 weeks after the quit date (OR = 1.85; 95% CI = 1.20~2.84).

**Conclusions:**

The SASEQ appeared to be a short, reliable, and valid questionnaire to assess self-efficacy beliefs regarding smoking abstinence. In the present study, this instrument also had good predictive validity. The short SASEQ can easily be used in busy clinical practice to guide smoking cessation interventions.

## Introduction

Self-efficacy is defined as the confidence a person has in his or her ability to perform and sustain a certain behavior in a given situation [1, 2]. It is an important component of several theories of behavior change. Efficacy expectations are proposed to be better predictors of behavior than are previous or current behaviors alone [3]. Self-efficacy depends on past experience with the behavior, influence of others, physiological state, and outcome expectations [4]. The concept of self-efficacy is particularly relevant for smoking cessation. People with a high confidence in their ability to quit smoking are more often successful in smoking cessation [5–7] and relapse less often after a quit attempt [8]. As self-efficacy is an important psychological construct with immediate relevancy and practical implications to smoking cessation, it is useful to measure it in routine clinical practice, for example in pregnant women or in heart patients.

In the past, various questionnaires to measure self-efficacy with regard to smoking cessation have been used [8–15]. These questionnaires generally consist of a list of smoking situations for which respondents can rate their confidence in their ability to refrain from smoking [16]. However, these instruments are not always feasible for use in routine clinical care as they are composed of twelve to 48 items. Therefore, a new, six-item self-efficacy scale was constructed: the Smoking Abstinence Self-efficacy Questionnaire (SASEQ).

In the current study, we investigate the psychometric properties of this six-item self-efficacy scale in a prospective smoking-cessation trial.

As self-efficacy may be influenced by motivation to quit smoking and depression [17–20], we investigated the association of self-efficacy with measures of motivation and depression. Self-efficacy is a separate concept from depression and motivation to quit smoking, so we hypothesized that correlations between these concepts would be low. Furthermore, we hypothesized that high scores on the SASEQ would predict smoking abstinence at 52 weeks after the quit date.

## Methods

### Participants

Between January 2004 and January 2007, 513 smokers participated in a smoking cessation program (STOPPERS). They were recruited by general practitioners of 15 general practices and specialists of the two departments of Máxima Medical Centre hospital in Eindhoven and Veldhoven. The inclusion criteria were willingness to discuss smoking behavior and sufficient understanding of the Dutch language. The only exclusion criterion was any participant suffering from a severe psychiatric disorder in immediate need of treatment.

### Procedure

All smokers received smoking cessation advice from their general practitioner or medical specialist. When patients showed interest in smoking cessation, they were referred to the study project. All participants were from Eindhoven and its surrounding areas, in the South East of the Netherlands. They all signed informed consent forms. The study protocol was approved by the Maxima Medical Centre ethics committee, which is certified by the Central Committee on Research involving Human Subjects in the Netherlands.

Participants were asked to complete several questionnaires. The quit date was set usually approximately 4 weeks after the inclusion in the study. Fifty-two weeks after the quit date, smoking status was assessed.

### Measures

#### Smoking Abstinence Self-efficacy Questionnaire

The SASEQ was constructed based on extensive experience with smoking cessation interventions and knowledge of the literature [8, 14, 15, 21–26]. The eight-item self-efficacy subscale as developed by Dijkstra, de Vries, and Roijackers [24] was used as a basic and further refined. It consists of two dimensions: four items describing “social” situations and four items describing “emotional” situations. Based on face validity two items were removed, one item: “going out with friends,” because it describes more or less the same situation as in the item: “being in a café or at a party,” and another emotional item (“feeling bored”) because it is quite different from the other emotional items: agitated, angry, and sad [24]. The remaining six items describe situations for which smokers can indicate on a 5-point Likert scale (0–4) whether they will be able not to smoke (Appendix I). The higher the score, the higher the level of self-efficacy regarding smoking cessation is. The range of the SASEQ scale is 0–24.

#### Edinburgh Depression Scale

The Edinburgh Depression Scale (EDS) [27–30] is a ten-item self-report scale which measures depressive symptoms. Respondents can rate on a 4-point Likert scale (0–3) to what extent they have had depressive feelings and thoughts over the past 7 days. The higher the score, the more depressive symptoms the respondent has. The range of the EDS is 0–30.

#### Symptom Checklist List-90 anxiety subscale

Anxiety was assessed by means of the anxiety subscale of the Symptom Checklist List-90 (SCL-90). The SCL-90 is used to assess psychopathology and has extensively been validated in the Netherlands [31]. The anxiety subscale consists of ten items that can be rated on a 5-point Likert scale [1–5]. The higher the score, the more anxious the respondent is. The range of the anxiety subscale is 10–60.

#### Motivation to Quit Smoking

Motivation to quit smoking was assessed with the following question “How motivated are you to quit smoking completely?” This question is derived from questionnaires of the MAYO Clinics in the USA. Respondents were presented with a 5-point Likert scale: (0) not at all motivated, (1) not very motivated, (2) neutral, (3) a little motivated, and (4) very motivated.

Demographic characteristics of the participants and smoking habits were also registered.

#### Smoking Status

Smoking status was assessed by self-report. At 52 weeks after the quit date, participants were asked if they had smoked since the original date of quitting. Long-term abstinence was defined as abstinence for at least 6 months. When participants did not provide follow-up data, we assumed they had started smoking again.

### Analyses

Explorative factor analysis was used to identify the underlying factors of the questionnaire. We used the principal axis factoring method with Varimax rotation. Prior to this analysis, the Kaiser–Meyer–Olkin measure of sampling adequacy and the Bartlett’s test of sphericity were examined to evaluate whether the data fulfilled the assumptions for carrying out a factor analysis. The Kaiser–Guttman criterion (eigenvalue > 1) was utilized to decide on the number of factors retained. Homogeneity of factor solution(s) was determined by calculating item-total correlations and internal consistency by Cronbach’s alpha. An alpha of ≥0.7 was regarded as sufficient [32].

The discriminant validity of the SASEQ was investigated by calculating Pearson correlations with the SCL anxiety subscale, the EDS, and motivation to quit smoking.

To determine the predictive validity of the SASEQ scale as a predictor of successful smoking cessation, we used logistic regression analysis. Successful smoking cessation was defined as not having smoked for the past six months. In the regression analysis, we also included known predictors for smoking cessation [33] in order to determine the role of the SASEQ score. We included the following predictors: gender, duration of longest quit attempt, smoking status of partner, average number of cigarettes per day, and duration of being a smoker. We also conducted a *t* test to see whether people who had achieved long-term abstinence at 52 weeks, scored higher on the SASEQ at baseline.

## Results

### Participants

Demographic characteristics are summarized in Table 1. The sample consisted of 52% women. The mean age was 51 years (SD = 11). Most participants had completed medium level education and were married or living with a partner. They smoked on average 20 cigarettes/day (SD = 10). The mean age when they smoked their first cigarette was 15 (SD = 3.35), and the mean age when they started smoking daily was 17 (SD = 4.19). Participants had undertaken on average 3.6 quit attempts (SD = 4.24). The average score on the item regarding their motivation to quit smoking was 3.4 (SD = 0.9); the mean SASEQ score was 11.7 (SD = 5.5).

**Table 1:** Characteristics of the study participants (*N* = 513)

	Mean (SD) or percentage	Range
Women	52%	
Age	51 (10.75)	21–78
Lower education	29.8%	
Medium education	49.5%	
Higher education	20.7%	
Married/living with partner	73.7%	
Single	26.3%	
Number of cigarettes/day	19.74 (10.17)	1–85
Age of first cigarette	15 (3.35)	6–40
Age when started smoking daily	17 (4.19)	10–50
Number of previous quit attempts	3.60 (4.24)	0–40
SASEQ total score	11.74 (5.48)	0–24
Depression	6.30 (5.20)	0–25
Motivation to quit	3.41 (0.84)	0–4

### Factor Analysis

The Kaiser–Meyer–Olkin measure (0.86) and Bartlett’s test of sphericity (*p* < 0.001) indicated that the assumptions for factor analysis were met. Exploratory factor analysis yielded one factor (eigenvalue > 1), with an eigenvalue of 3.8, explaining 64% of the variance. The second factor had an eigenvalue of 0.79; therefore, it is not taken into account. All factor loadings were ≥0.73 (Table 2). The factor structure was the same for men and women and across different educational levels.

**Table 2:** Factor loadings of SASEQ

	Loading
You feel agitated or tense	0.74
You are (very) angry	0.73
You are in a café, at a party, or paying a visit	0.79
You feel (very) sad	0.78
Someone offers you a cigarette of your own brand	0.74
You see someone enjoy smoking	0.75

Eigenvalue, 3.83 and percentage of explained variance, 63.86

### Internal Consistency

The internal consistency of the SASEQ was good, we found a Cronbach’s alpha of 0.89, and if items were deleted, Cronbach’s alpha decreased. Item-total correlations for items 1–6 ranged between 0.68 and 0.73.

### Discriminant and Predictive Validity

We found significant very low and negative correlations for the SASEQ with the EDS depression score (*r* = −0.145; *p* = 0.001). Furthermore, we found a low, positive, significant correlation for the SASEQ with motivation to quit (*r* = 0.205; *p* < 0.001).

The logistic regression analysis was conducted with smoking status as the dependent variable, and with self-efficacy, gender, duration of longest quit attempt, smoking status of partner, average number of cigarettes per day, and duration of being a smoker as covariates.

We found that only the SASEQ score significantly predicted smoking status at 52 weeks after the quit date. Participants with higher scores on self-efficacy were significantly less likely to start smoking again (OR = 0.95; 95% CI = 0.91~0.99; *p* = 0.02). We also conducted a *t* test to see whether people who had achieved long-term abstinence at 52 weeks, scored higher on the SASEQ. We found that non-smokers at 52 weeks indeed had significantly higher SASEQ self-efficacy scores (*t* = 2.68; *df* = 511; *p* = 0.008): the mean SASEQ score for smokers was 11.41 (SD = 5.41); whereas the mean SASEQ score for non-smokers was 13.00 (SD = 5.60).

## Discussion

This study investigated the psychometric properties of a six-item self-efficacy scale for smoking abstinence. Factor analysis of the SASEQ showed one factor with an explained variance of 64%. All factor loadings were adequate. The SASEQ had high internal consistency (Cronbach’s alpha = 0.89) and good discriminant validity. We found a significant, very low, negative correlation for the SASEQ with depression, and a significant, low, positive correlation with motivation to quit smoking. These findings support the disciriminant validity of SASEQ, indicating that this instrument does not measure depression or motivation to quit smoking, and confirms that self-efficacy is indeed a separate concept from these two concepts.

To investigate the predictive validity of the SASEQ, we analyzed whether our respondents' SASEQ scores predicted smoking status. We found that the SASEQ score before the planned quit date significantly predicted smoking abstinence at 52 weeks after the quit date. The odds ratio of 1.85 indicates that people who score high on the SASEQ, have a much higher chance to abstain from smoking, compared with people who score low on the SASEQ (95% CI = 1.20~2.84). Furthermore, we found that non-smokers at 52 weeks had rated themselves significantly higher on self-efficacy before quitting.

Our results indicate that the SASEQ is a very good questionnaire for use in a clinical setting. It is psychometrically sound and very short: with only six items, it can be easily completed in a waiting-room, or incorporated in a larger questionnaire booklet without adding too many extra questions. It should be noticed that in the Netherlands, nowadays, quit smoking strategies have been implemented in large chronic health care programs (diabetes, cardiovascular risk management, and COPD), managed by GP nurses in Primary Care [34]. Unfortunately, within these health care programs and in contrast to the assessment of concepts as depression and anxiety, appropriate instruments are lacking to detect the patient’s characteristics with regard to capability of changing behavior. This is not only important for quit smoking strategies but also in motivating diabetics for improving daily activities or obese patients to change their eating behavior. Because the GP nurse is often confronted with chronic patients with a high degree of co-morbidity and the outpatient clinic consultation time is limited, short instruments are needed which can easily be used in daily practice. Moreover, when reliable instruments do exist which are able to discriminate between patients with high and low self-efficacy, it might be speculated that—in view of cost-effectiveness—in the future different programs of different intensity can be offered to different patients.

A limitation of the study is that there were no other self-efficacy measures available to correlate the SASEQ score with, in order to assess convergent validity. Another limitation is the fact that motivation to quit smoking was measured with one item, instead of making use of a questionnaire. Strong points of the study are the prospective design and the large sample size.

In conclusion, the SASEQ seems to be an instrument that can assess self-efficacy regarding smoking abstinence reliably and validly. SASEQ scores appeared to be significant predictors of successful smoking cessation. We would like to emphasize that this six-item questionnaire can be completed in approximately 1 min and is therefore feasible for use in busy clinical practice.

## Appendix 1: The Smoking Abstinence Self-efficacy Questionnaire (SASEQ)

1. You feel agitated or tense. Are you confident that you will not smoke?

□ Certainly

□ Probably

□ Neutral / don’t know

□ Probably not

□ Certainly not

2. You are (very) angry. Are you confident that you will not smoke?

□ Certainly

□ Probably

□ Neutral/don’t know

□ Probably not

□ Certainly not

3. You are in a café, at a party, or paying a visit. Are you confident that you will not smoke?

□ Certainly

□ Probably

□ Neutral/don’t know

□ Probably not

□ Certainly not

4. You feel (very) sad. Are you confident that you will not smoke?

□ Certainly

□ Probably

□ Neutral/don’t know

□ Probably not

□ Certainly not

5. Someone offers you a cigarette of your own brand. Are you confident that you will not smoke?

□ Certainly

□ Probably

□ Neutral/don’t know

□ Probably not

□ Certainly not

6. You see someone enjoy smoking. Are you confident that you will not smoke?

□ Certainly

□ Probably

□ Neutral/don’t know

□ Probably not

□ Certainly not

The scores for the subsequent responses are the following:

Certainly = 4

Probably = 3

Neutral/don’t know = 2

Probably not = 1

Certainly not = 0
